# Computational identifying and characterizing circular RNAs and their associated genes in hepatocellular carcinoma

**DOI:** 10.1371/journal.pone.0174436

**Published:** 2017-03-27

**Authors:** Yan Li, Yongcheng Dong, Ziyan Huang, Qifan Kuang, Yiming Wu, Yizhou Li, Menglong Li

**Affiliations:** 1 College of Chemistry, Sichuan University, Chengdu, China; 2 College of Life Science, Sichuan University, Chengdu, China; University of North Carolina at Chapel Hill School of Medicine, UNITED STATES

## Abstract

Hepatocellular carcinoma (HCC) is currently still a major factor leading to death, lacking of reliable biomarkers. Therefore, deep understanding the pathogenesis for HCC is of great importance. The emergence of circular RNA (circRNA) provides a new way to study the pathogenesis of human disease. Here, we employed the prediction tool to identify circRNAs based on RNA-seq data. Then, to investigate the biological function of the circRNA, the candidate circRNAs were associated with the protein-coding genes (PCGs) by GREAT. We found significant candidate circRNAs expression alterations between normal and tumor samples. Additionally, the PCGs associated with these candidate circRNAs were also found have discriminative expression patterns between normal and tumor samples. The enrichment analysis illustrated that these PCGs were predominantly enriched for liver/cardiovascular-related diseases such as atherosclerosis, myocardial ischemia and coronary heart disease, and participated in various metabolic processes. Together, a further network analysis indicated that these PCGs play important roles in the regulatory and the PPI network. Finally, we built a classification model to distinguish normal and tumor samples by using candidate circRNAs and their associated genes, respectively. Both of them obtained satisfactory results (~ 0.99 of AUC for circRNA and PCG). Our findings suggested that the circRNA could be a critical factor in HCC, providing a useful resource to explore the pathogenesis of HCC.

## Introduction

HCC is the most common type of liver cancer and the third most common causes of cancer worldwide [1, 2]. The majority of HCC arise from chronic hepatocellular B virus infection and subsequent cirrhosis [3]. Its characteristics of rapid development and early metastasis lead to a poor prognosis, making the prevention of HCC remains a formidable task, a major problem is lack of reliable biomarkers. It is therefore necessary to explore its regulatory processes for understanding its pathogenesis.

CircRNAs as a new type of non-coding RNA are distinct from traditional linear RNAs for their closed loop structure. They are widely presented in the eukaryotic transcriptome [4]. CircRNAs are predominantly generated by back splicing of exons in eukaryotic genomes [5, 6]. These RNA have been reported abundant, conserved and stable in cytoplasm and could play specific roles as microRNA sponges, regulation of transcription, protein binding and translation into protein and potential anti-cancer effects [4–9]. However, only a few such circRNAs have been well interpreted [10, 11], and identification of circRNAs is still a challenging task both in bioinformatics and experiments [12]. The biological function of the majority of the circRNAs and their regulatory mechanisms remain largely unknown.

Previous studies showed that circRNAs associate with disease occurrence [6]. CircRNAs were found in hepatitis delta virus, which result in severe liver disease [13]. Particularly, circHIPK3 was reported significantly overexpression in liver cancer tissues compared with normal tissues [10]. The expression pattern of circRNAs in cancer serum from colorectal cancer patients was significantly different from normal serum that some were missing or detected in cancer patients [14]. These studies indicated that the aberrant circRNA expression may be a critical factor for the pathogenesis of diseases. However, the circRNA expression profiling has so far been poorly addressed relative to PCGs.

In this work, we investigated whether aberrant expression of circRNAs and their related PCGs associated with HCC. The main work flow is as follows: firstly, two algorithms, circRNA_finder [15] and circExplorer [16], were used to annotate circRNAs for a more reliable output due to pairing any two algorithms could greatly decrease false positive rates [17]. We focused on the common outputs by the two algorithms as the candidate circRNAs. Further, to examine the biological function of these candidate circRNAs, they were associated with PCGs by using the Genomic Regions Enrichment of Annotations Tool (GREAT) [18]. The enrichment analysis of these PCGs, the MSigDB perturbation [19] showed that the PCGs associated with these circRNAs displayed aberrant expression in HCC. And a further disease ontology [20] analysis illustrated they are significant enriched on the live/ cardiovascular-related disease. The GO analysis [21] showed that they significantly participate in metabolic-related process. The clustering analysis showed that both the candidate circRNAs and their associated PCGs displayed different expression patterns between the normal and tumor samples. Additionally, protein-protein interaction (PPI) network analysis suggested that these PCGs exhibit significantly different topology properties than other genes in the PPI network. We then constructed a circRNA-microRNA-gene regulatory network, in which, the genes predominantly enriched on metabolic and regulation-related process and participate in cancer-related pathway. Finally, classification models were built to classify normal and tumor samples by using circRNAs and their associated PCGs, respectively. In both cases, the normal samples could be accurately distinguished from the tumor samples. These results showed that the formation of circRNAs may relate to HCC occurrence.

## Materials and methods

### Data

RNA-seq data of 100 samples consisting of tumor tissues and para-carcinoma tissues from 50 HCC patients were downloaded from the Sequence Reads Archive (SRP068976) [22]. And the corresponding expression data of all genes in each sample also was downloaded. All of these 50 paired normal and tumor samples were paired-end sequenced using standard Illumina protocols and total RNA molecules were extracted. For the clinical information of patients see S1 Table. The human reference genome (hg19) was downloaded from UCSC Genome Browser (http://genome.ucsc.edu/). STAR (version 2.5.2a) and Bowtie (version 2.2.8.0/1.2.2.0) were used to build index with default parameters for the human genome.

### Identification and quantification of circRNAs

Recently, circRNA attracted widespread attention, and several tools were developed for annotating circRNAs by exploiting RNA-sequencing reads spanning the back-splicing junction. According to previous study [17], there exist dramatic differences between the circRNAs annotation algorithms, but pairing up any two algorithms would greatly decrease the false positive rate and produce a more reliable output. Particularly, combining any two algorithms could result in 8–12% false positive fraction, while ~6.6% by combining all the five algorithms. As the tradeoff the computation time and false positives, two algorithms, circExplorer and circRNA_finder algorithms were combined used for circRNA annotation. The circExplorer requires gene annotation information which is more reliable and more time-consuming. The circRNA_finder is a *de novo* prediction tool which is unbiased and faster. For each algorithm, the prediction outputs of all the samples were dealt with the python scripts provided by *Thomas B*. *Hansen* [17]. Then the circRNAs validated by both these two algorithms were kept for the following analysis. The averaged read counts spanning the back splice junctions by the two algorithms were used as a measure of the expression level.

### Associating circRNAs with PCGs by GREAT

We adopted only the common candidate circRNAs by the two algorithms to decrease the false positive rates based on previous reports by [17]. By this way we obtained 2091 circRNAs. Most of the circRNAs were made up of exons that defined as exonic circRNA. According to previous studies circularization through back-splicing could make the aberrant expression of its host gene [4, 23]. To further estimate the function of these candidate circRNAs, the GREAT (version 3.0.0) [18] was employed to associate circRNAs with their nearby PCGs. The gene regulatory domains were defined by using default basal plus extension (Proximal: 5kb upstream, 1kb downstream, plus Distal: up to 1000 kb).

### The random forest classification model

Machine learning algorithms are well accepted for classification [24, 25]. To investigate whether the expression of circRNAs relate to HCC occurrence, we applied random forest to distinguish tumor samples from normal samples [26] by using the transcriptional profile of circRNAs/genes as input. The total 2091 common circRNAs by both algorithms were reserved as the candidates for model features. In addition, we also built a classification model by using the PCGs associated with the circRNAs which were also required had significant expression alterations between normal and tumor samples (t-test, p value < 0.01).

In practice, the random forest was applied to make our prediction with default 500 trees. 80 samples were randomly selected as the training data and the remaining 20 samples as the testing data. This process was repeated 100 times. Five-fold Cross-validation was used to model training. To avoid a biased inflation of predictability, we performed feature selection against the training data by using the LASSO [27] According to the ten-fold cross-validation MSE, the optimized lambda was got by minimizing the MSE. The value of alpha is always kept at the default value of 1. Lasso could set a coefficient of 0 to the less informative features and the remains with non-zero coefficients were then used as the input to a random forest classifier.

## Results

### PCGs associated with circRNAs play critical role in HCC

CircRNA is a new type of non-coding RNA, attracting widespread attention in recent years. Previous studies showed that circRNA displayed the anomalism in disease, could regard as a promising biomarker for disease diagnosis and targeted therapy. However, only a few circRNAs are well interpreted, and the biological function of the majority of circRNAs remains unclear. A systematically investigation for circRNAs expression is therefore important to explore its function and regulatory mechanisms.

**Fig 1:**
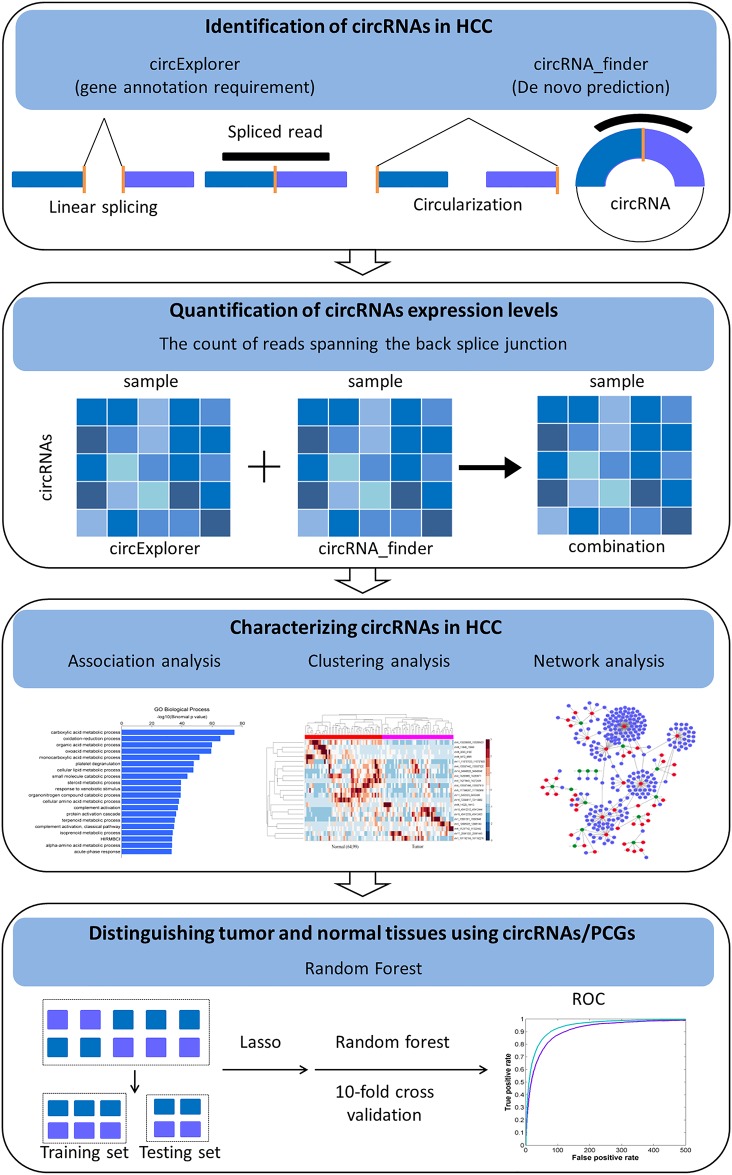
Flow chart of the key stages in our analysis pipeline.

Here, the 2091 common candidate circRNAs (S2 Table) identified by these two algorithms were kept for the following analysis. The workflow was shown in Fig 1. To validate our predictions we searched against the available public circRNA database. Among them, 47% has been reported by previous studies recorded in the circNet database [28]. For example, circHIPK3 (chr11:33307958–33309057) from HIPK3 exon2 was found display aberrant expression in liver compared with matched normal tissues, and its silence could inhabit human cell proliferation [10].

**Fig 2:**
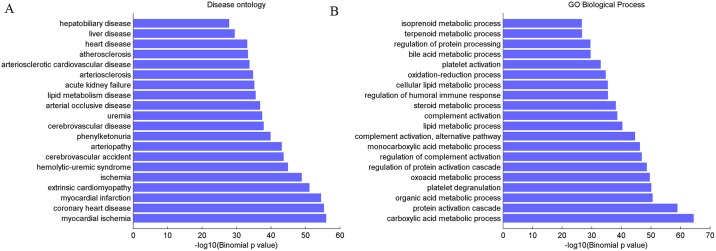
Enrichment analysis of PCGs associated with aberrantly expressed circRNAs by using GREAT. (A) Top 20 disease terms were found significantly enriched on liver and cardiovascular-related diseases (B) top 20 GO terms with predominantly involved in metabolic-related process.

To examine the potential biological functions of the candidate circRNAs, these 2091 candidate circRNAs were associated with their nearby PCGs by GREAT (details see Methods). Interestingly, we found that these PCGs not only enriched on liver/hepatobiliary disease but also enriched on cardiovascular-related disease, such as atherosclerosis, myocardial ischemia and coronary heart disease *etc*. (Fig 2A). According to the previous studies, it may be due to the strong correlation of liver disease and cardiovascular disease [29–31]. And a further GO enrichment analysis [21] (Fig 2B.) suggested that these PCGs significantly participate in metabolic-related process such as oxidation-reduction metabolic process and organic acid metabolic process, which in line with previous reports that tumor tissue exhibit a remarkably different metabolism [32]. In the case of HCC, the abnormal organic acid metabolism has reported by [33]. Additionally, MSigDB Perturbation ontology analysis [19] (Table 1), with well-defined gene signatures of genetic and chemical perturbations, suggested that these PCGs closely related to HCC. Expectively, these PCGs are characterized by the increased proliferation, high levels of serum AFP, which is an important mark for HCC diagnosis [34]. All these results suggested that the formation of circRNAs likely related with HCC occurrence.

**Table 1:** The MSigDB perturbation enrichment result for PCGs associated with aberrantly expressed circRNAs.

MSigDB Perturbation Term Name	Binom Raw P-Value	Binom FDR Q-Val	Binom Fold Enrichment
**Liver selective genes**	1.30E-165	4.38E-162	7.617453
**Genes from 'subtype S3' signature of hepatocellular carcinoma (HCC): hepatocyte differentiation**.	8.61E-117	1.45E-113	5.527136
**Genes down-regulated at early fetal liver stage (embryonic days E11.5—E12.5) compared to the late fetal liver stage (embryonic days E14.5—E16.5)**.	3.90E-75	4.37E-72	5.37416
**Genes down-regulated in hepatocellular carcinoma (HCC) compared to normal liver samples**.	1.30E-63	1.10E-60	3.241819
**Genes negatively correlated with recurrence free survival in patients with hepatitis B-related (HBV) hepatocellular carcinoma (HCC)**.	3.25E-56	2.19E-53	8.102399
**Top 200 marker genes down-regulated in the 'proliferation' subclass of hepatocellular carcinoma (HCC); characterized by increased proliferation, high levels of serum AFP [Gene ID = 174], and chromosomal instability**.	1.70E-48	9.55E-46	4.658689

### CircRNAs and their associated PCGs are significantly altered between tumor and normal samples

**Fig 3:**
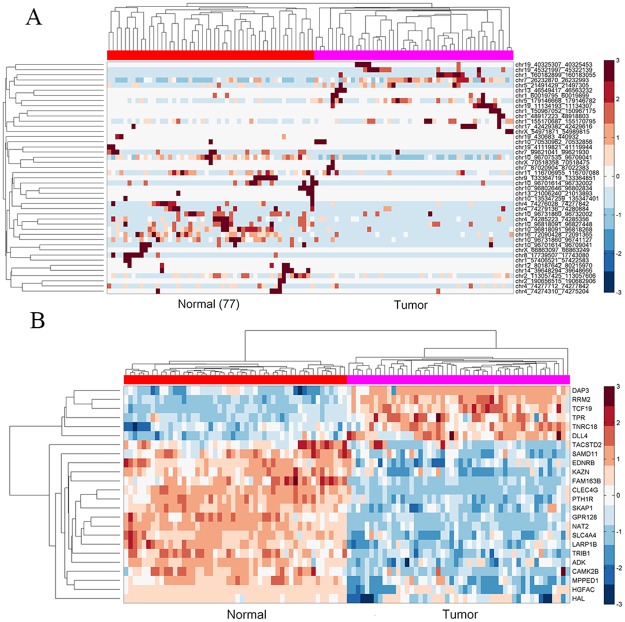
Comparison analysis of circRNAs/PCGs expression profiles. (A) 45 critical circRNAs displayed expression difference between normal and tumor samples. (B) 23 PCGs displayed expression difference between normal and tumor samples. The clustering analyses were performed in MATLAB using clustergram function (columnPDist: correlation).

To further investigate the expression pattern of candidate circRNAs in HCC, a clustering analysis was performed. By a feature selection of LASSO [27], 45 circRNAs were extracted with significant contribution for discriminating tumor samples from normal samples. The normal and tumor samples could be accurately separated by these circRNAs (only one sample was wrongly assigned) (Fig 3A). There are 34 PCGs associated with them. We used these PCGs to perform clustering analysis, found they also display significantly different expression between normal and tumor samples.

Secondly, we focused on the 2622 PCGs associated with the 2091 candidate circRNAs (details see Methods). T-test analysis was performed on these PCGs, 1450 (~55%) PCGs were detected differentially expressed between normal and tumor samples (p value < 0.01) and kept for subsequent analysis. Similarly, a feature selection by LASSO was carried on this gene set. 23 PCGs were identified exhibiting significant contribution for discriminate normal and tumor samples. We found the expression profile of these 23 PCGs could accurately distinguish normal and tumor samples by clustering analysis (Fig 3B).

### Network analysis of critical circRNAs and PCGs in protein-protein network and circRNA-microRNA-gene regulatory network

**Fig 4:**
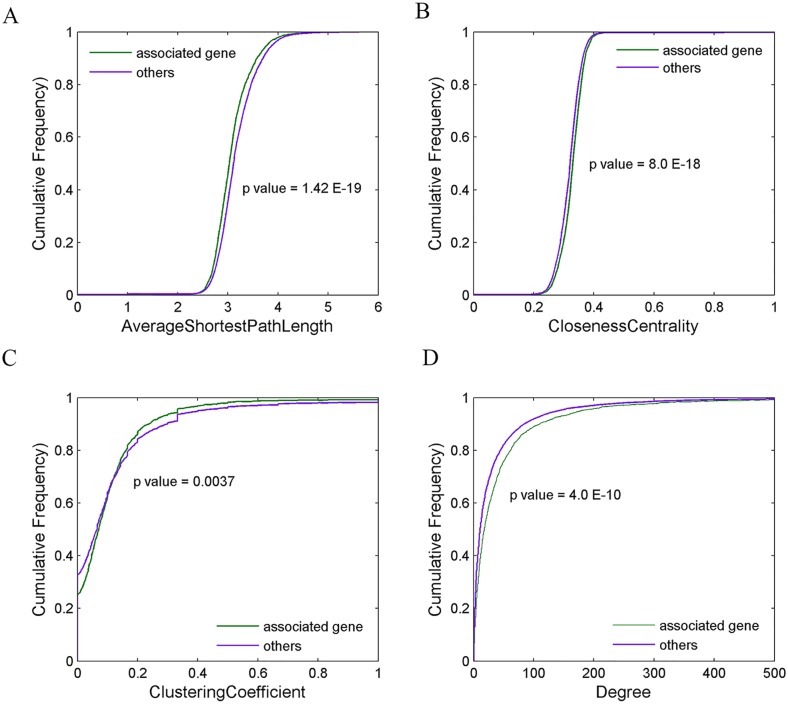
Cumulative distributions of topology properties in the PPI network. (A) Average shortest path length (B) Closeness centrality (C) Between coefficient (D) Degree. Purple represents the PCGs associated with circRNAs, and green represents other PCGs in the network.

In order to further understand the characteristic of the circRNAs, we made an analysis on their topological characteristics in the network. First, we analyzed the topological properties of genes associated with circRNAs in protein-protein network (PPI) downloaded from BIOGRID database [35]. Interestingly, the topological properties were significantly different between the PCGs associated with circRNAs and others. The PCGs associated with circRNAs displayed higher degree and closeness centrality and lower average shortest path length and clustering coefficient (Fig 4, t-test), suggesting important roles of these circRNAs-related PCGs in the PPI.

**Fig 5:**
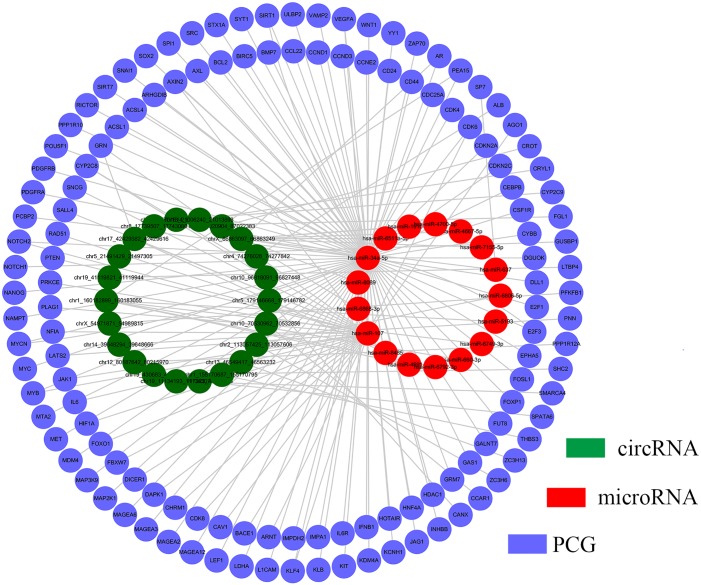
Network analyses of critical circRNAs and PCGs. The regulatory network of circRNA-microRNA-gene extracted from CircNet. The circRNAs were above used in clustering analysis (green), the microRNAs were directly interact with these circRNAs (red), and the PCGs were directly interact with these circRNAs or microRNAs (purple).

**Fig 6:**
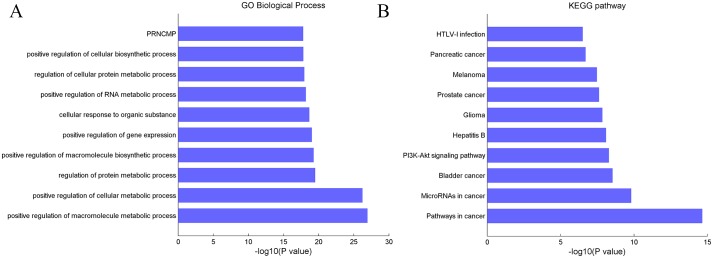
Enrichment analysis of PCGs in the regulatory network. (A) GO enrichment analysis. (B) KEGG pathway analysis. PRNCMP (positive regulation of nitrogen compound metabolic process).

To further examine the biological function of circRNAs, we focused on the targets of above mentioned 45 critical circRNAs in clustering analysis based on the circRNA-microRNA-gene regulatory network by [28], in which the potential miRNA binding sites on circRNAs were determined by iteratively searching well defined microRNA binding motif against the circRNA sequences. Among them, there were 22 circRNAs were also reported in the circNet. Then, we extracted their microRNA targets and also the gene targets for both these microRNAs and circRNAs. Finally, a circRNA-microRNA-gene regulatory network was constructed consisting of 22 circRNAs, 17 microRNAs and 130 PCGs (Fig 5, S3 Table). Among these microRNAs, several microRNAs were found related to cancers, such as has-miR-34a-5p has been reported related to several types of cancers, for example, colorectal cancer [36]; has-miR-107 exhibit aberrant expression in a variety of cancers, including hepatocellular [37], gastric cancer [38], and Glioma [39] etc. The GO analysis suggested that these PCGs mainly involved in metabolic and regulation-related process (Fig 6A). The KEGG pathway analysis showed that these PCGs predominantly participate in cancer-related process (Fig 6B), including Hepatitis B. Furthermore, t-test analysis suggested that the majority of these PCGs are differentially expressed between tumor and normal samples (~63%, p value < 0.01). This result implies that the circRNAs could function in diversity ways to influence the gene expression.

### CircRNAs and their associated PCGs could accurately distinguish normal from tumor samples

According to above clustering analysis, circRNAs and their associated PCGs were significantly altered between normal and tumor samples. In order to further examine their expression patterns, we built a classification model to investigate whether the circRNAs or their associated PCGs has the ability to distinguish normal and tumor samples, respectively.

Firstly, the expression levels of the candidate circRNAs across all the samples were applied as the feature vectors inputting to random forest classifier. Specifically, we randomly selected 80 samples used as the training data, and the remaining samples were used as the testing data. Feature selection only used in training data to avoid biased inflation of predictability. This process was repeated 100 times, and the average values of predictive results were listed in Table 2. Our result suggested that circRNA could successfully distinguish tumor and normal samples, and the resulting AUC was 0.988 for the training data and 0.976 for the testing data (Table 2). The result suggests that circRNA is strongly correlated with HCC. In addition, the 34 PCGs (PCG set 1) directly associated with the 45 circRNAs used in clustering analysis were also applied to distinguish normal and tumor samples, and obtained a high accuracy (Table 2).

**Table 2:** The prediction performance of circRNAs or PCGs.

		Acc	Sen	Spe	Pre	Mcc	AUC	AUPR
**CircRNA**	**Training Data**	0.940	0.936	0.943	0.944	0.880	0.988	0.989
	**Testing Data**	0.903	0.900	0.914	0.912	0.811	0.976	0.977
**PCG set 1**	**Training Data**	0.945	0.948	0.941	0.942	0.890	0.988	0.989
	**Testing Data**	0.936	0.949	0.928	0.930	0.876	0.986	0.987
**PCG set 2**	**Training Data**	0.992	0.984	1.000	1.000	0.984	1.000	1.000
	**Testing Data**	0.983	0.967	1.000	1.000	0.967	0.997	0.998

We also built a classification model by using aforementioned 1450 differentially expressed PCGs that associated with 2091 circRNAs. Expectedly, the prediction accuracy is satisfactory (PCG set 2, Table 2), suggesting that employing circRNA to select critical genes is indeed an efficient method, and the changes of circRNAs may be closely related to hepatocarcinogenesis.

## Discussion

A large number of publicly available RNA-seq data with high sequencing depth in normal and tumor tissues allow us to investigate the genetic state of a tissue. The circRNAs prediction tool contributed to explore the pathogenesis of HCC from the perspective of circRNAs. To our knowledge, many of circRNAs don’t encode for proteins, but rather a fast-growing set of transcripts were discovered [4]. The new class of non-coding RNA is increasingly being proposed to play critical roles in a variety of diseases and become a promising potential biomarker for cancer diagnosis [18]. Although, a few circRNAs has been shown to be shared microRNA binding sites as the competitive endogenous RNA (ceRNA), acting as microRNA sponges [40, 41]. However, the regulation of circRNA expression has so far been poorly addressed relative to PCGs. It is remains unclear for most of the circRNAs biological functions.

In this work, circRNAs were identified based on 50 paired normal and tumor HCC RNA-seq data. 2091 circRNAs were identified by both algorithms. The PCGs relevant to them display important regulatory roles in liver/ cardiovascular-related disease, such as atherosclerosis, myocardial ischemia and coronary heart disease. GO and MsigDB perturbation analysis also showed that these PCGs were closely to liver occurrences. Additionally, network analysis suggested that these PCGs were critical factors in the regulatory and PPI network. In addition, there exist significant expression differences of some circRNAs their associated PCGs between normal and tumor tissue in HCC. Both of them could accurately distinguish tumor and normal samples, likely correlated with HCC occurrence. These findings provide a new resource to investigate the HCC pathogenesis, contributing to improve the HCC diagnosis and therapy.

To this study, we found there are individual differences of the circRNAs expression. Here some of circRNAs were detected at only several patients. Aberrant expression analysis and network inference based on a group of samples with similar phenotype, which prevents disease diagnosis on one sample from one individual. So, it may be valuable to study the post-transcriptional regulation process of circRNA from the perspective of personalization. Due to the insufficient of clinical information, we couldn’t perform more specific analysis combined with phenotype of patients. We would develop deep-depth analysis in future work.

## Conclusions

In this study, 50 paired normal and tumor HCC RNA-seq data were employed to investigate the roles of circRNAs in HCC. The PCGs associated with the 2091 circRNAs were found enriched on liver/cardiovascular-related diseases. Furthermore, 45 circRNAs and 23 PCGs were identified, displaying significant expression differences between normal and tumor tissues, respectively. The classification model suggested that expression profiles of the both two gene types could accurately distinguish normal and tumor samples. These results contribute to our understanding of the underling pathologic mechanism.

## Supporting information

S1 Table The clinical information of patients. (XLSX)

S2 Table The identified candidate circRNAs by both the circExplorer and the circRNA_finder. (XLSX)

S3 Table The edges in the circRNA-microRNA-gene regulatory network. (XLSX)
